# Denture Care Behavior and Lifespan of Removable Partial Dentures in Indonesian Military Personnel: An Approach Using the Theory of Planned Behavior

**DOI:** 10.1055/s-0044-1800827

**Published:** 2024-12-30

**Authors:** Nining Handayani, Arief Budiarto, Arif Rachman, Arlette Suzy Setiawan

**Affiliations:** 1 Postgraduate Doctoral Study Program, Military Dentistry Faculty of Dentistry Universitas Padjadjaran, Bandung West Java, Indonesia; 2 Departement of Psychology, Universitas Jenderal Ahmad Yani, Bandung West Java, Indonesia; 3 Departement of Cell Biology and Biomolecular, Faculty of Medicine, Indonesian Defense University, Bogor West Java, Indonesia; 4 Department of Pediatric Dentistry, Faculty of Dentistry, Universitas Padjadjaran, Bandung, West Java, Indonesia

**Keywords:** partial removable dentures, soldiers, lifespan, maintenance behavior, oral health

## Abstract

**Objectives** In the context of military health, removable partial acrylic dentures (RPADs) are crucial for the quality of life and performance of Indonesian National Armed Forces (TNI) soldiers. Given the demanding and unpredictable military environment, RPADs provide a solution for soldiers experiencing tooth loss. This research aims to identify behavioral factors influencing the lifespan of RPADs among TNI soldiers.

**Materials and Methods** This study employed an observational cross-sectional design with an analytical approach. The study population consisted of all patients fitted with RPADs at Ladokgi RE Martadinata from 2017 to 2019. The inclusion criteria included TNI soldiers using good functionality RPADs from 2017 to 2019. Data were collected through a valid questionnaire (Cronbach's alpha = 0.738) and analyzed using Spearman's rank correlation and multiple linear regression.

**Results** In total, 46 respondents (84.8% males, aged 50–59 years) participated in this study. The average behavioral score was 153.72, with a standard deviation of 1.13. The average lifespan of RPADs was 5.33 years. The correlation analysis showed a moderate positive correlation between behavioral scores and RPAD lifespan ( *r* = 0.463, *p* = 0.001). Multiple regression analysis indicated a significant association between behavioral scores and RPAD lifespan, with a coefficient *B* -value of 0.259 ( *p* = 0.012).

**Conclusion** Good RPAD maintenance behavior correlates with a longer RPAD lifespan. Socialization programs and instructions for RPAD care need enhancement to ensure optimal quality of life and performance among TNI soldiers.

## Introduction

In the context of military health, the maintenance of dental prostheses is a crucial aspect that contributes to the quality of life and performance of the Indonesian National Armed Forces (TNI) soldiers. 1 Removable partial acrylic dentures (RPADs) serve as a solution for soldiers who have experienced tooth loss, 2 considering the often demanding and unpredictable military environment, with soldiers potentially moving around and being in the field for extended periods. RPADs provide a durable and adaptable solution for this mobile and frequently changing lifestyle. 3 4

Proper care of RPADs not only affects comfort and eating functionality but also prevents long-term complications that could disrupt these soldiers' duties. Routine maintenance, including daily cleaning of RPADs and soaking them in a cleaning solution when not in use, is essential in maintaining the hygiene and durability of the dentures. 2 5 6 7 However, it is crucial to understand and address the challenges, such as the demands of duty and lack of access to dental care facilities in the field, that can affect soldiers' compliance with denture maintenance.

With soldiers' increasing use of dentures, there has been a noted increase in cases requiring RPAD repairs at the Ladokgi TNI AL RE Martadinata (REM) Dental and Oral Hospital. Data show that RPAD users account for 68% of the patients treated, with the majority of these soldiers needing denture repair interventions, evidenced by 48.1% of them undergoing control with the purpose of denture repair.

This study aims to identify behavioral factors that influence the lifespan of RPADs in the TNI service environment. It hopes the results will provide insights for improving the quality of dental and oral health services within the military environment.

## Materials and Methods

This observational study, which uses an analytical approach and a cross-sectional design, has obtained approval from the Research Ethics Committee of Universitas Padjadjaran with document number 168/UN6.KEP/EC/2023. The study population includes all patients fitted with RPADs at Ladokgi REM from 2017 to 2019. The target population for the study consists of all patients who have RPADs installed, and the accessible population includes patients who visited Ladokgi REM and met the following criteria: TNI soldiers who had RPADs installed at Ladokgi REM, started using RPADs from 2017 to 2019, and RPADs that are still in good functional condition. Patients who have repaired their RPADs within the past 3 years, have systemic diseases like diabetes mellitus, or take medications affecting saliva flow (such as hypertension drugs) are excluded from the study. We excluded patients who repaired their RPADs within the past 3 years, those with systemic diseases such as diabetes mellitus and those taking medications affecting saliva flow because these factors can directly influence denture longevity. For example, systemic conditions like diabetes and medications affecting saliva flow could impact the oral environment, increasing the risk of denture failure. Our aim was to control for these confounders to isolate the behavioral factors related to denture care.

The study selected a significance level of 5% ( *Zα* = 1.96), a power test of 80% ( *Zβ* = 0.84), and a clinically significant correlation coefficient of *r* = 0.4. Based on these parameters, a minimum of 46.7 participants are required for the study. We rounded down the calculation to 46 participants.

The behavioral factors studied are actions taken by patients in caring for their RPADs, precisely (attitude toward targeted behavior, subjective norms, perceived behavioral control, intention, and past behavior) according to Ajzen's theory. 8 The denture care behavior questionnaire consists of 22 items designed based on the theory of planned behavior (TPB), involving all five domains of TPB. The questionnaire has been validated through testing with 30 respondents, achieving a Cronbach's alpha value of 0.738, indicating good internal consistency. The questionnaire is self-administered, and the researcher monitors the completion.

**Attitude toward the behavior** reflects the extent to which someone holds positive or negative evaluations regarding denture care behavior. This attitude is shaped by individuals' beliefs about the outcomes of performing that behavior and their evaluations. Attitude assessment related to respondents' opinions about “soaking dentures in a container of room temperature water when not in use” and “cleaning dentures daily with running water and brushing them with a toothbrush.” Attitudes were measured using a three-word statement in a semantic differential format. The direct assessment involved the use of bipolar adjectives. These questions about attitudes used a 7-point Likert scale, such as 1 = detrimental to 7 = beneficial, 1 = troublesome to 7 = relieving, and 1 = boring to 7 = enjoyable. The total score for respondents' attitudes was constructed by summing six items (ranging from 6 to 42). A higher score indicated a more positive attitude.

**Subjective norms** refer to an individual's perception of social pressure to perform or not perform denture care behaviors. This perception includes beliefs about the views of significant others (such as family, friends, and colleagues) regarding whether the individual should engage in these denture care behaviors. Subjective norm assessment contained questions like “What do you think about always reminding/telling me to soak dentures in a container of room temperature water when not in use?” and “What is your opinion about cleaning dentures under running water and brushing them daily?” Subjective norms were evaluated using six items based on reference groups or individuals closest to the respondent and who tend to exert social pressure regarding the behavior to motivate the respondent (known individuals and denture users). These subjective norms used a 7-point Likert scale (1 = disagree to 7 = agree). Total subjective norm scores were calculated by summing the scores of six items (ranging from 6 to 42). A higher score indicated higher positive subjective norms.

**Perceived behavior control** reflects an individual's belief about the ease or difficulty of performing denture care behaviors. This belief is influenced by past experiences and anticipation of barriers or supports in performing the behavior. Questions concerning perceived behavioral control included items on how confident the respondent is to perform the target behaviors (soaking dentures in a container of room temperature water and cleaning dentures under running water) and the difficulty or ease of doing so. Perceived behavioral control was measured using a sum score constructed from six items (9–14), rated on a 7-point Likert scale (1 = disagree to 7 = agree). The sum score for respondents' perceived behavioral control was calculated by adding the scores of the six items (ranging from 6 to 42). A higher score indicated more positive perceived behavioral control.

**The intention** is to determine whether someone will engage in denture care behaviors. Attitudes toward the behavior, subjective norms, and perceived behavioral control influence this intention. The stronger someone's intention, the more likely they will perform the behavior. Intention can also be used as a measure of proximal behavior. The intention was evaluated with two questions: “Do you intend to soak dentures in a container of room temperature water routinely?” and “Do you intend to clean dentures under running water and brush them?” The intention score was obtained by summing the scores of these two items. Respondents indicated their intention on a 7-point scale, where 1 = no intention and 7 = firm intention (ranging from 7 to 14). A higher score indicated a more positive intention.

**Past behavior** is the actual actions performed by individuals in the past few months up to the day of the study. In the TPB, behavior is directly influenced by intention and indirectly influenced by perceived behavioral control. Behavior in this study is reflected in questions about behavior over the past 3 months. Two questions were the following: “In the past 3 months, have you soaked dentures in a container of room temperature water when not in use?” and “In the past 3 months, have you cleaned dentures under running water and brushed them daily?” The behavior score was obtained by summing the scores of these two items. Respondents indicated their behavior on a 7-point scale, where 1 = disagree to 7 = agree (ranging from 7 to 14). A higher score indicated higher positive past behavior.

**Lifespan** of denture use refers to the length of time respondents have used their dentures from insertion until the date of the study. The duration data are recorded in years, rounded up to the nearest whole year.

Collected data are processed and analyzed descriptively and analytically. The normality of numeric data are tested using the Shapiro–Wilk test; data are normally distributed if the obtained *p* -value is less than 0.05. Correlation analysis is conducted using Spearman's rank correlation if the data are not normally distributed. The dominant influence between independent and control variables on the dependent variable is analyzed using multiple linear regression. The significance of test results is determined based on *p* -values less than 0.05. All data processing and analysis are performed using SPSS version 26.

## Result

**Fig. 1:**
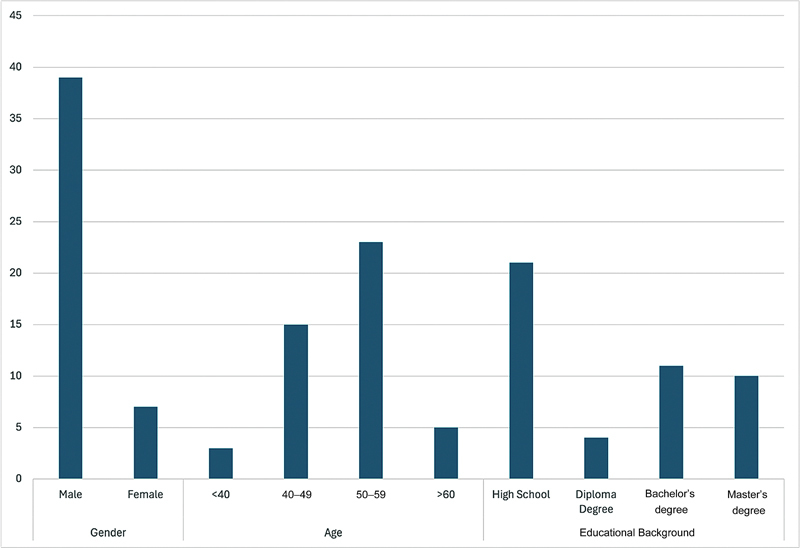
Respondent characteristics.

Fig. 1 illustrates the characteristics of the 46 respondents in this study. The gender is predominantly male (39 respondents/84.8%), with 23 respondents (50%) aged between 50 and 59 years. The highest level of education completed is high school for 21 individuals, accounting for 45.7% of the respondents. All participants enrolled in the study had removable partial dentures (RPDs) in a single arch. Specifically, 28 participants (60.9%) had maxillary RPDs, while 18 participants (39.1%) had mandibular RPDs. The number of teeth replaced by each denture ranged from two to three. These details provide insight into the types of dentures used by the participants and contribute to understanding the factors influencing denture longevity.

Table 1 presents the research results on aspects of behavior and the duration of RPADs. This table includes several behavior variables: attitude toward behavior, subjective norms, perceived behavioral control, intention, and past behavior, as well as the duration of RPDs in years.

**Table 1:** Results on behavioral aspects and the lifespan of denture

Variable	Mean	SD	Data normality test ( *p* -value) *
Behavior			
● Attitude toward behavior ● Subjective norms ● Perceived behavior ● Intention ● Past behavior	153.72 41.9 41.9 41.8 13.9	1.13 0.29 0.29 0.61 0.14	<0.001
Duration of RPD use (y)	5.33	0.82	<0.001

Abbreviations: RPD, removable partial denture; SD, standard deviation.

Note: Past behavior is constant, it has been omitted.

* Based on the Shapiro–Wilk test.

For behavior variables, the average score is 153.72, with a standard deviation of 1.13. Specifically, attitude toward behavior averages 41.9, with a standard deviation of 0.29. Subjective norms also have an average of 41.9 with similar values for standard deviation, median, and range. Perceived behavioral control shows an average of 41.8, with a standard deviation of 0.61. The intention variable has an average of 13.9, with a standard deviation of 0.14. The past behavior variable is constant and therefore excluded from detailed analysis.

The average lifespan of RPADs is 5.33 years, with a standard deviation of 0.82, a median of 6 years, and a range of 4 to 6 years. Normality tests using the Shapiro–Wilk test resulted in *p* -values less than 0.001 for all variables, indicating that the data are not normally distributed. These results indicate significant variation and deviation from normal distribution for all measured variables.

[ Table 1 ]

Table 2 presents the correlation between behavior scores and duration of RPD use. The Spearman's rank correlation coefficient ( *r* ) is 0.463, indicating a moderate positive correlation between these two variables. This correlation suggests that as the behavior scores increase, the duration of RPD use also increases. The *p* -value is 0.001, which is highly significant, implying that the observed correlation is statistically significant and not due to random chance. Thus, there is a meaningful relationship between higher behavior scores and longer duration of RPD use among the respondents.

**Table 2:** Correlation between behavior scores and the duration of removable partial denture use

	Correlation coefficient ( *r* )	*p* -value
Behavior scores and duration of removable partial denture use	0.463	0.001

Note: *r* = Spearman's rank correlation.

Table 3 presents a multiple regression analysis examining the relationship between behavior scores and duration of RPAD use. In the initial model, the coefficient *B* for behavior scores is 0.253, with a standard error (S.E.) of 0.098. The *t* -value is 2.598, and the *p* -value is 0.013. These results indicate that behavior scores have a significant positive relationship with the duration of denture use, suggesting that higher behavior scores are associated with longer durations of use.

**Table 3:** Relationship between behavior scores and the duration of removable acrylic partial denture use based on multiple regression analysis

Variable	Coefficient *B*	SE ( *B* )	*t* -value	*p* -value
Initial model				
Behavior scale	0.253	0.098	2.598	0.013
**Final model**				
Behavior scale Constant	0.259 –33.450	0.099 –	2.612 –	0.012

Abbreviation: SE, standard error.

Note: For the final model, *F* = 6.689 ( *p* = 0.003) with multiple *R* ^2^ = 0.202.

In the final model, the coefficient *B* for behavior scores slightly increases to 0.259, with a S.E. of 0.099. The *t* -value is 2.612, and the *p* -value is 0.012, confirming the significance of the relationship. The constant is –33.450, although its standard error and *t* -value are not provided. The final model's overall fit is indicated by an *F* -value of 6.689 ( *p* = 0.003), demonstrating that the model is statistically significant. The multiple *R* ^2^ value is 0.202, meaning that the behavior scores explain approximately 20.2% of the variance in the duration of denture use. This model highlights a significant positive relationship between behavior scores and duration of RPAD use.

## Discussion

The research findings indicate that male respondents outnumber female respondents. This disparity is likely due to the predominance of males in the military, which may have influenced the respondent demographics. Moreover, tooth loss, regardless of gender, can be attributed to oromaxillary trauma commonly experienced by military personnel as part of their duties. Oromaxillofacial trauma is commonly experienced by military personnel due to their physical activities and duties. This result aligns with studies such as Rachinsky et al, which explain that military conflicts lead to mass disabilities, including dental disabilities and fatalities, resulting in a predominantly male military personnel population engaged in combat operations. 9 A research in Bosnia found that 17.91% of military personnel required tooth extraction due to aging. 10 This aligns with the research findings that show that individuals aged 50 to 59 years are dominantly affected by tooth loss as they age.

Military physical activities that lead to oromaxillofacial trauma often result in tooth loss, which is replaced by either fixed or removable acrylic dentures. Acrylic dentures, whether fixed or removable, are dental prostheses designed to replace missing teeth, restoring chewing function and aesthetics for patients. 11 Mouth rehabilitation with dentures significantly impacts daily life and has tremendous social implications, helping restore confidence for regular interaction. 12

Behavior scores positively influence the lifespan of RPADs, while nonbehavior scores negatively impact their lifespan. Higher behavior scores tend to extend the lifespan of RPADs, whereas lower nonbehavior scores indicate more extended durability. The analysis shows that the combined model of behavior and nonbehavior scores correlates with the lifespan of RPADs. Environmental factors influence oral health by approximately 40%, while behavioral factors influence by about 30%. Together, environmental and behavioral factors contribute to over two-thirds of oral health status in the community, particularly in behaviors related to cleaning natural teeth and dentures. 13 Attitudes toward obtaining health are an innovation that can motivate respondents. Health programs and socialization efforts, along with instructions on denture care, can encourage research subjects to adopt positive values related to oral health maintenance, leading to improved knowledge and attitudes. 14

Using the TPB, this study also analyzed various behavioral variables influencing the lifespan of RPADs among TNI soldiers. These variables include attitude toward behavior, subjective norm, perceived behavioral control, intention, and past behavior. 15

Attitude toward behavior reflects how positively or negatively someone evaluates the behavior of denture care. 15 16 In this study, attitudes were assessed based on individuals' beliefs about the outcomes of performing these behaviors and their evaluations. Positive attitudes, such as “soaking dentures in room temperature water when not in use” and “cleaning dentures daily with running water and brushing them with a toothbrush,” indicate that respondents understand the importance of these actions for maintaining good denture hygiene. The research findings indicate that a more positive attitude toward denture care correlates with a longer lifespan.

Subjective norm refers to an individual's perception of social pressure to perform or not perform denture care behaviors. 17 This includes beliefs about the views of significant others (such as family, friends, and colleagues) regarding whether the individual should engage in those behaviors. 18 A high subjective norm indicates that respondents feel supported by their social environment to take good care of their dentures. 17 18 19 This study found that strong social support contributes to better denture care behaviors, ultimately extending the lifespan of dentures.

Perceived behavioral control reflects an individual's beliefs about the ease or difficulty of performing denture care behaviors. It is influenced by past experiences and the anticipation of barriers or support in carrying out these behaviors. 20 In this study, perceived behavioral control includes beliefs about the ability to soak dentures and clean them daily. The results indicate that higher perceived behavioral control leads to better denture care among respondents, correlating with a longer lifespan.

Intention is a crucial factor in determining whether someone will engage in denture care behavior. It is influenced by attitude toward the behavior, subjective norms, and perceived behavioral control. The stronger someone intends to care for their dentures, the more likely they are to perform that behavior. 17 18 20 This study found that solid intention correlates with better denture care practices, contributing to a longer denture lifespan.

Previous behavior refers to the actual actions taken by individuals in caring for their dentures over a specific period. 21 In this study, past behavior was evaluated based on the frequency of denture care actions over the last 3 months. The results showed that the dentures of respondents who consistently soaked and cleaned them had a longer lifespan. Good past behavior reflects established habits that reinforce positive care practices. 21 22 23

## Limitations of the Study

This study has several limitations that need to be considered. First, the population involved only includes TNI soldiers visiting Ladokgi REM, so the results may not be generalizable to all TNI soldiers or other military populations. Second, the cross-sectional study design only allows for observation of relationships between variables at a single point in time and cannot determine causal relationships. Third, data on denture care behaviors were collected through self-reported questionnaires, which may be influenced by respondent bias or inaccuracies in memory. Fourth, the study may not have considered all variables that could affect denture lifespan, such as environmental conditions at duty stations, access to dental health services, and variations in the quality of dentures used. Fifth, external factors such as dental health policies in the military environment, levels of dental health awareness and education among soldiers, and support from colleagues or superiors were not evaluated in this study. Sixth, the study only covered a 3-year denture lifespan, so longer durations might provide a more comprehensive understanding of denture resilience and influencing factors. Finally, the study did not measure the direct effects of care behaviors on overall oral health conditions, focusing solely on denture lifespan. Therefore, drawing conclusions and applying the study's findings to broader populations should be done cautiously.

One potential limitation of this study is the environmental constraints faced by military personnel, which may affect their ability to adhere to proper denture care routines. Soldiers may be deployed in remote or hostile environments, where regular denture maintenance could be challenging. For instance, during maneuvers or extended periods in the field, access to necessary facilities or time for personal care may be limited. These factors could influence the effectiveness of self-management and care of RPDs. Future research should consider these deployment conditions when evaluating denture care practices in military settings.

## Conclusion

This study demonstrates that acrylic partial denture care behavior among TNI soldiers significantly influences the lifespan of these dentures. Higher behavior scores correlate positively with longer lifespan, emphasizing the importance of proper denture maintenance in extending their usability. Factors such as attitude toward care, subjective norms, perceived behavioral control, and intention to care for dentures all play crucial roles in determining the success of acrylic partial denture use. However, the study also highlights the need to address limitations such as a limited population, cross-sectional study design, and unexamined external factors. Therefore, efforts to enhance education and support for denture care in military settings are crucial for improving the quality of life and performance of TNI soldiers.
